# International Practice Variability in Treatment of Aneurysmal Subarachnoid Hemorrhage

**DOI:** 10.3390/jcm10040762

**Published:** 2021-02-14

**Authors:** Jordi de Winkel, Mathieu van der Jagt, Hester F. Lingsma, Bob Roozenbeek, Eusebia Calvillo, Sherry H-Y. Chou, Peter H. Dziedzic, Nima Etminan, Judy Huang, Nerissa U. Ko, Robert Loch MacDonald, Renee L. Martin, Niteesh R. Potu, Chethan P. Venkatasubba Rao, Mervyn D. I. Vergouwen, Jose I. Suarez

**Affiliations:** 1 Department of Neurology, Erasmus MC, University Medical Center Rotterdam, 3000 CA Rotterdam, The Netherlands; j.dewinkel@erasmusmc.nl (J.d.W.); b.roozenbeek@erasmusmc.nl (B.R.); 2 Department of Public Health, Erasmus MC, University Medical Center Rotterdam, 3000 CA Rotterdam, The Netherlands; h.lingsma@erasmusmc.nl; 3 Department of Intensive Care Adults, Erasmus MC, University Medical Center Rotterdam, 3000 CA Rotterdam, The Netherlands; m.vanderjagt@erasmusmc.nl; 4 Department of Neurology, The Johns Hopkins University School of Medicine, Baltimore, MD 21287, USA; ecalvil2@jhmi.edu (E.C.); phd@jhu.edu (P.H.D.); npotu1@jhmi.edu (N.R.P.); 5 Departments of Critical Care Medicine, Neurology, and Neurosurgery, School of Medicine, University of Pittsburgh, Pittsburgh, PA 15260, USA; hsc36@pitt.edu; 6 Department of Neurosurgery, University of Heidelberg School of Medicine, 69117 Mannheim, Germany; nima.etminan@medma.uni-heidelberg.de; 7 Department of Neurosurgery, The Johns Hopkins University School of Medicine, Baltimore, MD 21287, USA; jhuang24@jhmi.edu; 8 Department of Neurology, UCSF Weill Institute for Neurosciences, UCSF School of Medicine, San Francisco, CA 94143, USA; nerissa.ko@ucsf.edu; 9 UCSF Fresno Department of Neurosurgery, UCSF School of Medicine, University Neuroscience Institute, Fresno, CA 93701, USA; rlochmacdonald@gmail.com; 10 Department of Biostatistics and Epidemiology, Medical University of South Carolina, Charleston, SC 29425, USA; hebertrl@musc.edu; 11 Departments of Neurology, Neurosurgery, and Center for Space medicine, Baylor College of Medicine, Houston, TX 77030, USA; cprao@bcm.edu; 12 Department of Neurology and Neurosurgery, UMC Utrecht Brain Center, University Medical Center Utrecht, Utrecht University, 3508 GA Utrecht, The Netherlands; m.d.i.vergouwen@umcutrecht.nl; 13 Division of Neurosciences Critical Care, Departments of Anesthesiology and Critical Care Medicine, Neurology, and Neurosurgery, The Johns Hopkins University School of Medicine, Baltimore, MD 21287, USA

**Keywords:** subarachnoid hemorrhage, outcome, practice variation, aneurysm treatment, fluid management, delayed cerebral ischemia, vasospasm

## Abstract

Prior research suggests substantial between-center differences in functional outcome following aneurysmal subarachnoid hemorrhage (aSAH). One hypothesis is that these differences are due to practice variability. To characterize practice variability, we sent a survey to 230 centers, of which 145 (63%) responded. Survey respondents indicated that an estimated 65% of ruptured aneurysms were treated endovascularly. Sixty-five percent of aneurysms were treated within 24 h of symptom onset, 18% within 24–48 h, and eight percent within 48–72 h. Centers in the United States (US) and Europe (EU) treat aneurysms more often endovascularly (72% and 70% vs. 51%, respectively, US vs. other *p* < 0.001, and EU vs. other *p* < 0.01) and more often within 24 h (77% and 64% vs. 46%, respectively, US vs. other *p* < 0.001, EU vs. other *p* < 0.01) compared to other centers. Most centers aim for euvolemia (96%) by administrating intravenous fluids to 0 (53%) or +500 mL/day (41%) net fluid balance. Induced hypertension is more often used in US centers (100%) than in EU (87%, *p* < 0.05) and other centers (81%, *p* < 0.05), and endovascular therapies for cerebral vasospasm are used more often in US centers than in other centers (91% and 60%, respectively, *p* < 0.05). We observed significant practice variability in aSAH treatment worldwide. Future comparative effectiveness research studies are needed to investigate how practice variation leads to differences in functional outcome.

## 1. Introduction

Spontaneous aneurysmal subarachnoid hemorrhage (aSAH) is a neurological emergency that continues to cause high morbidity and case-fatality, leading to over 27% of all stroke-related years of potential life lost before the age of 65 and a very high cost to society [1,2,3,4,5,6,7,8]. Approximately one in three aSAH survivors are left dependent [9,10]. The most dreaded complications after aSAH include rebleeding, early brain injury (EBI), and delayed cerebral ischemia (DCI), which are the main causes of neurological deterioration and disability [11,12]. Despite advances in diagnosis, prevention, and treatment of complications of aSAH, only a modest improvement in outcome has been observed [5,9,11]. The main therapies that improve long-term clinical outcomes and that are supported by evidence from randomized controlled trials are endovascular repair of the ruptured cerebral aneurysm in cases where the aneurysm is amenable to either coiling or surgical clipping, and administration of oral nimodipine to decrease the risk of DCI [13]. Most other management is based on weak evidence. Prior retrospective observational and registry studies have suggested that there are substantial between-center differences in functional outcome following aSAH that are most likely explained by case volume and variabilities in care [14]. Understanding which variabilities in care have an impact on aSAH outcome represents an important and dire unmet need. In this survey study, we aimed to characterize variations in treatment and organizational aspects of care that may impact patient outcomes.

## 2. Materials and Methods

### 2.1. Survey Development

In 2019 a ‘’Provider Profiling Questionnaire’’ was sent to 230 Neurocritical Care Research Network (NCRN)-affiliated sites worldwide to recruit participants for the International Subarachnoid Hemorrhage Comparative Effectiveness Research Alliance (INSIDER) study (See: Electronic Supplementary File S1). INSIDER is a planned seven-year prospective observational study to determine practice variability in aSAH and its effect on outcome. The survey was developed based on the clinical expertise of the principal investigators. Various disciplines (neurologists, neurosurgeons, neuro-intensivists, and epidemiologists) participated in its development. The survey consisted of several topics covering elements of aSAH treatment, which have been the subject of debate in recent decades, such as the type and timing of aneurysm treatment, fluid management, and treatment of DCI. Both open-answer and multiple-choice questions were used. In the present survey study, we focus on the Questions 1, 5, 7, 9–12, 17–22, 24–25, 27–34, and 36. The Institutional Review Board (IRB) at the Johns Hopkins University School of Medicine approved the study protocol under the exemption category and waived the need for written, informed consent.

### 2.2. Statistical Analyses

Descriptive statistics were used to describe aSAH practice variability and displayed in tables or figures. Categorical variables were presented as frequencies and percentages. Continuous variables were presented as medians with interquartile ranges or means with standard deviations. A geographical map was used to represent participating centers and their countries of origin. For further analyses, centers were geographically categorized as European, United States (US), or non-European or non-US, henceforth called “other”. Chi-squared test and ANOVA were used to compare regional differences. Post-hoc multiple comparison was performed with Bonferroni test. Analyses were performed with IBM SPSS Statistics, Version 25, for Windows (IBM, Chicago, IL, USA) and open-source software RStudio, Version 3.6.3, for macOS (R Foundation for Statistical Computing, Vienna, Austria).

## 3. Results

### 3.1. Center Characteristics

**Figure 1:**
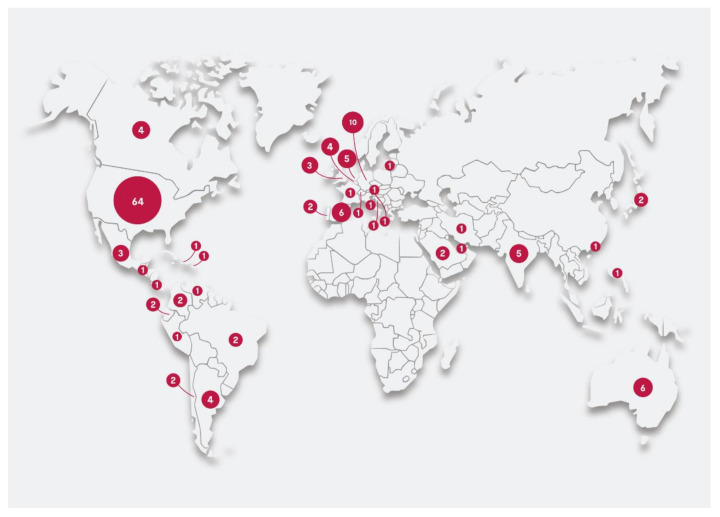
Geographical map representing the countries of origin of participating centers.

A total of 145 centers across five continents responded (response rate 63%). Of the survey respondents, 64 (44%) were located in the US (Figure 1 and Table 1), 37 (26%) were European, and 44 (30%) were from other areas.

The majority of centers were academic (*n* = 121, 84%). The number of beds varied greatly between centers. Most centers had 0–100 intensive care unit (ICU) beds (*n* = 112, 76%). The median number of neurological ICU beds was 15 (IQR 8–24). 

On average, 47% (SD 27) of patients with aSAH presented primarily to the survey respondents’ center. Most of the participating centers treated at least 200 aSAH patients per year (*n* = 73, 49%). aSAH patients were in most cases admitted to a dedicated neurological ICU (*n* = 96, 66%) or at a medical-surgical ICU (*n* = 38, 25%).

Completion rate of the survey’s questions varied from 48% (*n* = 69) to 100% (*n* = 145). Some proportions exceeded 100% because multiple answers were allowed.

### 3.2. Aneurysm Treatment

**Figure 2:**
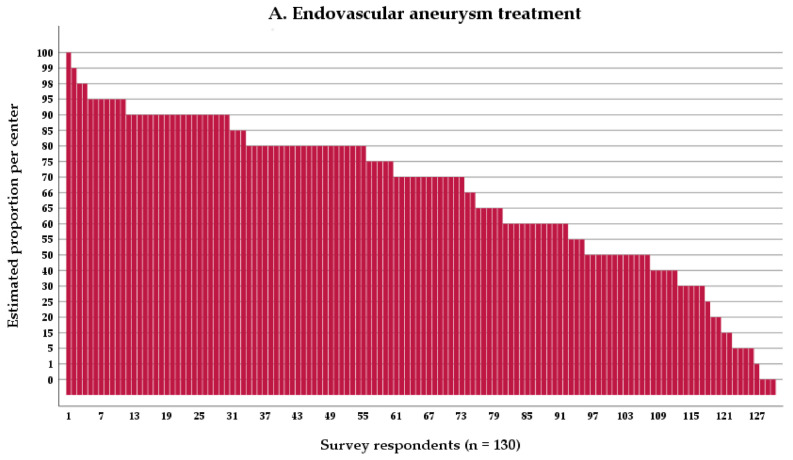

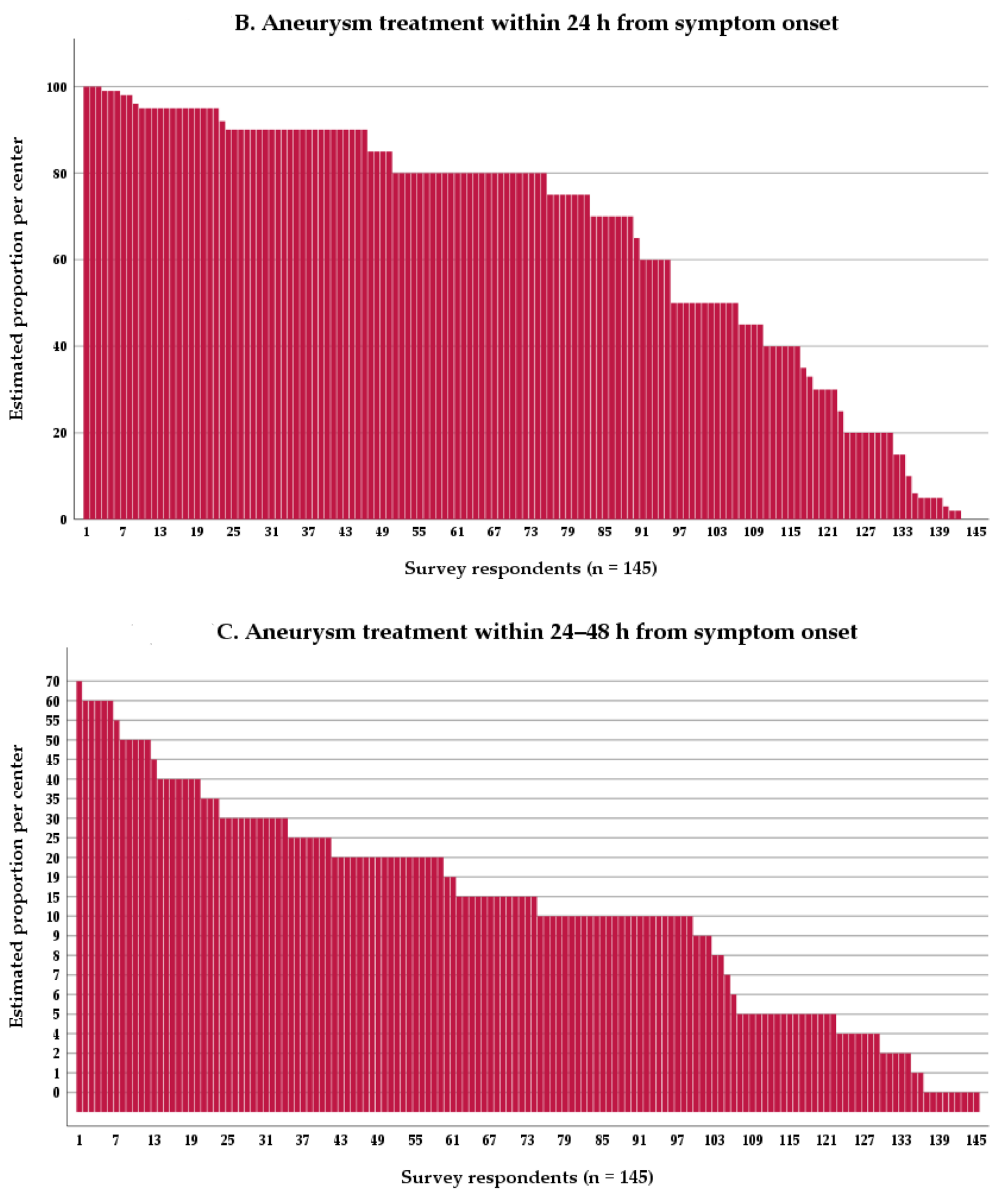

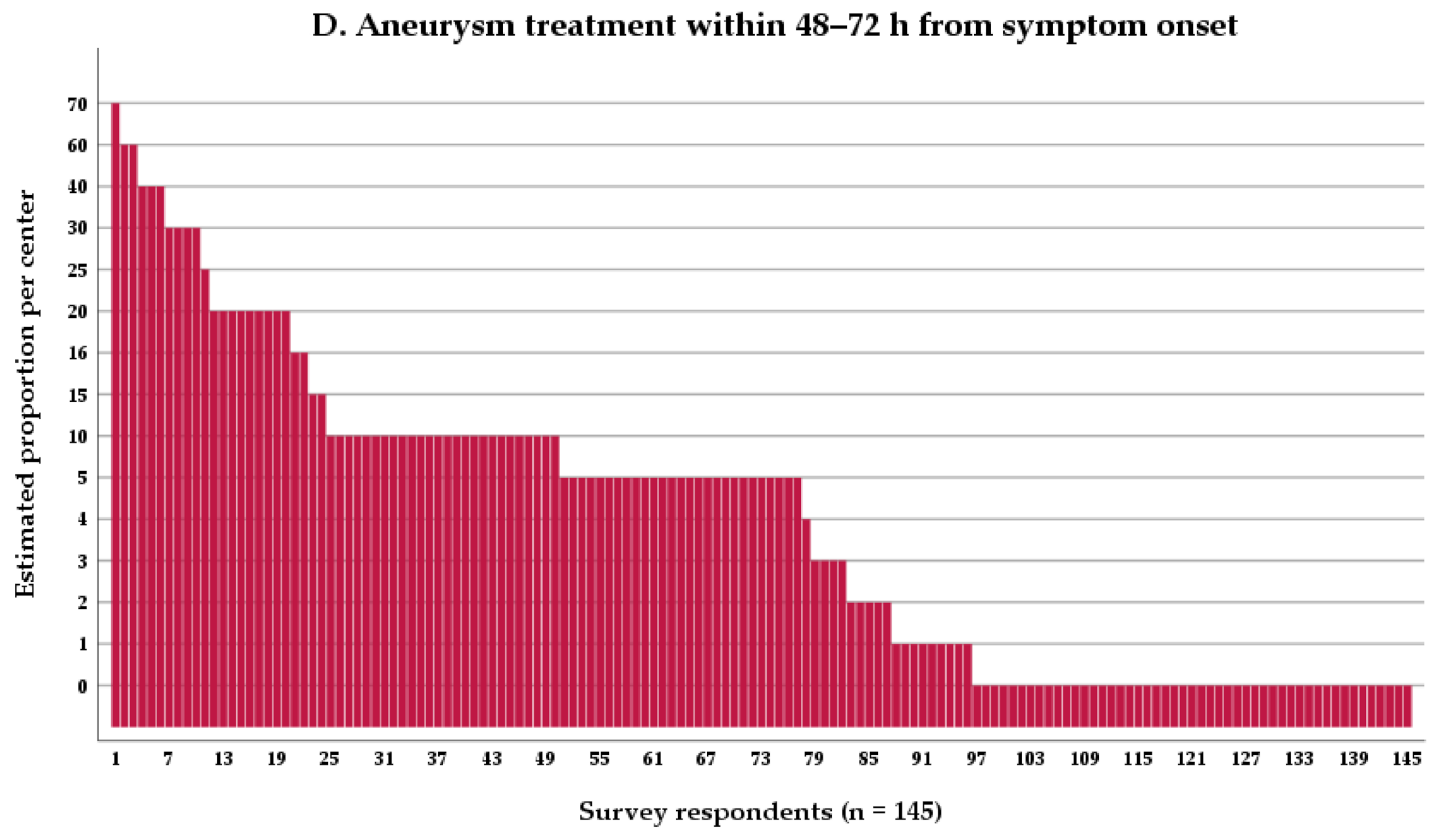
Type and timing of aneurysm treatment: (**A**) per center estimated proportion of patients with aneurysmal subarachnoid hemorrhage (aSAH) who have their aneurysm treated by endovascular means, (**B**) per center estimated proportion of patients with aSAH who have their aneurysm treated within 24 h from symptom onset, (**C**) per center estimated proportion of patients with aSAH who have their aneurysm treated within 24–48 h from symptom onset, and (**D**) per center estimated proportion of patients with aSAH who have their aneurysm treated within 48–72 h from symptom onset.

Overall, survey respondents indicated that a mean estimated 65% of all treated ruptured aneurysms were treated endovascularly. In addition, a mean estimated 65% of treated aneurysms were treated within 24 h of symptom onset, whereas a mean estimated 18% were treated within 24–48 h, a mean estimated 8% were treated within 48–72 h, and a mean estimated 9% of aneurysms were unaccounted for (Table 2, Figure 2A–D). It is unknown if the latter 9% accounts for aneurysms treated later than 72 h, not treated at all, or a combination of both. 

Ninety-six centers (66%) reported treating the majority (e.g., >50%) of aneurysms within 24 h (Figure 2B). Seven centers (5%) treat the majority of aneurysms between 24–48 h (Figure 2C), and only three (2%) 48–72 h after onset (Figure 2D). Thirty-nine centers (27%) do not treat the majority of aneurysms in any particular time window.

In US and European centers, aneurysms were more often treated by endovascular techniques than in other centers (Table 3, mean estimates 72%, 70%, and 51%, respectively; European vs. other *p* < 0.01; and US vs. other *p* < 0.001). The estimated proportion of aneurysms being treated within 24 h was equal in US and European centers but higher than in other centers (mean estimate 77%, 67%, 46%, respectively, European vs. other *p* < 0.01 and US vs. other *p* < 0.001).

### 3.3. Fluid Management

Nearly all centers aimed for a euvolemic state in aSAH patients in their hospital (*n* = 136, 96%, Table 4). Furthermore, 66 (53%) of survey respondents targeted for 0 mL/day net fluid balance and 51 (41%) for +500 mL/day, adjusting for insensible losses. None aimed for a negative net fluid balance. To reach the preferred volemic state, 66 (54%) respondents administered 2 L fluid daily, and 19 (16%) balanced this with output. The most commonly used maintenance fluid was 0.9% saline (*n* = 101, 86%) or, alternatively, balanced solutions (*n* = 59, 50.0%). Again, some proportions exceeded 100% as multiple answers were allowed. 

In centers where fluid management was guided by clinical blood testing (*n* = 69, 49%), the most common compounds measured were lactate (*n* = 56, 81%), B-type natriuretic peptide (*n* = 31, 45%), and troponin (*n* = 30, 44%). They were often assessed in some sort of combination (*n* = 37, 54%). When fluid management was guided by advanced hemodynamic monitoring in the ICU (*n* = 108, 84%), this was performed with echocardiography of inferior vena cava (*n* = 85, 77%), pulmonary artery catheter (*n* = 11, 10%), transpulmonary thermodilution (*n* = 53, 48%), or by other means (*n* = 29, 26%). Eighteen survey respondents (17%) estimated using advanced hemodynamic monitoring in less than 10% of aSAH patients, 40 (38%) in 10–25%, and 47 (45%) in more than 25%.

### 3.4. Cerebral Vasospasm and Delayed Cerebral Ischemia

**Figure 3:**
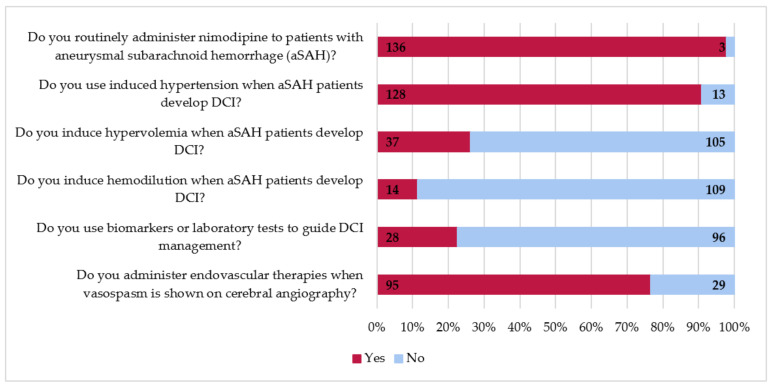
Management of delayed cerebral ischemia (DCI) in survey respondents’ hospitals.

Almost all centers routinely administer nimodipine in patients with aSAH (*n* = 136, 98%, Figure 3). Similarly, most centers induce hypertension if the patients develop DCI (*n* = 128, 91%). However, induced hypertension was more often used in US centers than European or other centers (Table 5, 100%, 87%, and 81%, respectively, US vs. other *p* < 0.05 and US vs. European *p* < 0.05). Less often, survey respondents indicated to use hypervolemia (*n* = 37, 26%) or hemodilution (*n* = 14, 11%) for treatment of DCI. About a quarter of survey respondents stated that they use biomarkers or laboratory testing to guide DCI management (*n* = 28, 23%). Endovascular treatment of angiographic vasospasm is commonly performed (*n* = 95, 77%), although significantly more in US centers than in European and other centers (91% vs. 74% and 60%, respectively; US vs. other *p* < 0.05).

## 4. Discussion

We performed an international and multi-center survey of hospital characteristics and treatment variation of aSAH patients. We observed significant variability of care of aSAH patients between individual centers as well as remarkable regional differences. Marked variability in treatment was observed in timing of aneurysm treatment, fluid management, and endovascular therapy of DCI. 

Our findings reaffirm a shift from neurosurgical clipping of ruptured aneurysms to endovascular aneurysm treatment. In our study, survey respondents estimated that 65% of patients were treated endovascularly. Contrarily, an earlier survey study reported only an estimated 37% of patients were treated endovascularly; however, this study was conducted more than a decade ago [15]. More recently, Velly et al. reported that 66% of survey respondents treated more than 60% of aneurysms by endovascular techniques [16]. Further analyses show that European and US centers treat aneurysms endovascularly equally as often, but significantly more often than centers in other parts of the world. Even though we did not collect data on factors that led to this shift in practice, it is important to point out that it may have been driven by results from important clinical trials [17,18]. The International Subarachnoid Hemorrhage Trial (ISAT) showed that when there is equipoise, the probability of disability-free survival is greater with endovascular coiling than with surgical clipping up to 10 years after treatment. The Barrow Ruptured Aneurysm Trial (BRAT) reported that outcome differences between these two treatment modalities may differ depending on aneurysm location, with better outcomes found in patients with posterior circulation aneurysms treated with endovascular coiling.

We found a strong preference for aneurysm repair within 24 h of the ictus. An estimated 65% of aneurysms were treated within 24 h from symptom onset. Additionally, we found that in European and US centers, aneurysms were equally as often treated within 24 h but significantly more often in comparison to centers in other geographic regions. Previous survey research found even greater proportions of aneurysm treatment within 24 h ranging from an estimated 79–81% [16,19]. However, both of these studies only included European respondents.

The American Stroke Association (ASA) guidelines recommend aneurysm treatment as early as feasible, and the European Stroke Organization (ESO) guidelines recommend repair as early as technically and logistically possible within 72 h after aSAH [1,20]. More recently, some experts advocated for aneurysm treatment on an emergency basis [21,22], primarily because rebleeding occurs most often in the hours following ictus [23,24,25,26,27]. Others found that aneurysm treatment within 24 h as opposed to 24–72 h was associated with worse outcome [28,29]. We were unable to investigate the relationship between timing of aneurysm treatment and outcome because our survey study did not collect individual patients’ outcomes.

One of the cornerstones of aSAH management is maintenance of euvolemia. We found that nearly all survey respondents (96%) aim for euvolemia as the goal of maintenance IV fluids. Euvolemia has been predicated on the observation that hypovolemia is associated with DCI [30]. As a result, current recommendations state that a negative fluid balance should be avoided in aSAH. In addition, more recent evidence indicates that hypervolemia may also be harmful [31,32].

To reach euvolemia, in many cases, survey respondents aim for a 0 mL/day (53%) or slight positive net fluid balance +500 mL/day (41%), adjusting for insensible losses. However, we observed significant variability in methods used to reach euvolemia: one-third of survey respondents did not clearly state a daily fluid intake goal (e.g., 1 L/day to >3.5 L/day) and about half of the respondents used clinical blood testing to guide fluid management despite questionable validity of such laboratory tests. Moreover, a great variety of clinical blood tests were used. Additionally, advanced hemodynamic monitoring was indicated to be widely used (84%), but it is unclear how this impacts fluid management. It is possible that this variation is explained by the less well-defined triggers to stop administering fluids. Optimizing fluid management strategies could potentially be an easy and affordable treatment target if supported by good quality evidence.

DCI is an important and much studied complication after aSAH, occurring in approximately 30% of patients [33]. Treatment of DCI includes hemodynamic and mechanical endovascular therapy or direct infusion of vasodilating drugs to reverse vasospasm. Despite a lack of evidence from randomized controlled trials to support use of any of these treatments and evidence for an increased risk of complications, our survey study showed that induced hypertension (91%), induced hypervolemia (26%), and induced hemodilution (11%) were frequently used. Interestingly, we observed an increased preference for using induced hypertension alone compared to previous survey research, which has found up to half of respondents use induced hypertension, hypervolemia, and hemodilution combined as treatment of DCI [15,19,34]. Our survey study underscores the variability in the management of DCI in patients with aSAH.

In the US, almost all aSAH patients (91%) are treated with endovascular techniques when vasospasm is shown on cerebral angiography, while this is significantly less used in other, non-European, parts of the world. Our results are in agreement with prior survey research that found comparable use of endovascular techniques among European survey respondents (78%) [16]. As expected, prevention of DCI with nimodipine (98%) was most widely accepted among our survey respondents.

An important strength of this survey study is that it offers insight into contemporary treatment variation. aSAH is a rapidly evolving field of medicine with notable improvements in intensive care management and aneurysm obliteration techniques. In addition to previous survey research, our study serves as a new worldwide benchmark for practice variability in aSAH and adds to other important prospective observational studies investigating the practice of treatment of neurocritically-ill patients in general [35,36].

Several limitations must be taken into account when interpreting this survey study. We included 145 centers with good worldwide representation, except the African continent, and an adequate response-rate of 65%. Nevertheless, there was an overrepresentation of US centers (44%), academic centers (84%), and centers located in high-income countries or areas (81%). Therefore, the results of this survey study might not be generalizable to centers located in low- and middle-income countries or non-academic centers. However, it is important to mention that aSAH patients are generally treated in specialized centers.

In our survey, we have asked to estimate the proportions of patients treated within 24 h, 24–48 h, and 48–72 h. We could not differentiate between aneurysms not treated at all and aneurysms treated later than 72 h (9% in total). A similar ambiguity is present in the question regarding proportions of aneurysms treated by endovascular or neurosurgical means. Although the question did not specifically ask for proportions of treated aneurysms, instead of all aSAH patients, we have interpreted the results as such. As mentioned above, some aneurysms remain untreated.

As in all survey-related research, this study is vulnerable to recall and responder bias. More specifically, the results of our survey study are based on the perception of aSAH center practice, not actual clinical practice. The latter will be most present in a minority of questions asking for estimated proportions. Other simple yes-or-no questions regarding clinical practice or specific center characteristics will most likely be less or unaffected by recall bias. However, we have not verified the accuracy of reported survey data. Additionally, possible bias may have been introduced because of incomplete responses to survey questions.

Furthermore, our survey participants included bedside clinicians, such as intensivists and neurosurgeons, and did not include neuroradiologists or neuro-interventionalists that do not participate in the daily care of aSAH patients. The addition of these practitioners’ perspectives in future surveys and studies would be valuable.

As randomized controlled trials are expensive, impractical, and sometimes ethically unjustifiable in the field of aSAH, there is an urgent call for other means to evaluate treatment outcomes in aSAH. Previous research suggests that significant between-center practice variability is associated with variation in clinical outcome [14]. We hypothesize that large practice variability can primarily occur in the absence of treatment strategies supported by high-quality evidence. This void leaves room for interpretation or personal preferences translating to practice variability. Future comparative effectiveness research (CER) should utilize this variability in aSAH care to determine whether it is associated with a clinical outcome.

In conclusion, we identified significant treatment variation in the type and timing of aneurysm treatment, fluid management, and endovascular therapies of DCI. We propose that future research focusses on these topics in relation to patient outcome as opportunities for CER.

## Figures and Tables

**Table 1:** Overview of center characteristics.

Center Characteristic	*n* Completed	*n* (%), Mean (SD) or Median (IQR)—Range
**Centers per region**	145	
United States		64 (44)
Europe		37 (26)
Other		44 (30)
**Type of institution**	145	
Academic		121 (84)
Private non-academic		11 (8)
Public non-academic		8 (6)
Other		5 (4)
**No. of hospital beds**	144	
<250		11 (8)
250–500		24 (17)
500–750		32 (22)
750–1000		41 (28)
>1000		36 (25)
**No. of intensive care unit (ICU) beds**	144	60 (33–100)—380
0–100		109 (76)
101–200		32 (22)
201–300		1 (1)
301–400		2 (1)
**No. of neurological ICU beds**	139	15 (8–24)—48
0–10		56 (39)
11–20		39 (27)
21–30		33 (23)
31–40		6 (4)
40>		4 (3)
**How many patients per year do you see with aneurysmal subarachnoid hemorrhage (aSAH) at your center?**	145	
0–40		5 (3)
40–60		25 (17)
60–100		17 (12)
100–150		12 (8)
150–200		14 (10)
>200		72 (50)
**What percentage of patients with aSAH present primarily to your hospital—as opposed to referred patients?**	142	47 (27)
0–25%		39 (27)
26–50%		50 (35)
51–75%		24 (17)
76–100%		29 (20)
**Where are aSAH patients admitted?** *	145	
Dedicated Neurological ICU		96 (66)
Surgical ICU		15 (10)
Medical ICU		9 (6)
Medical-Surgical ICU		36 (25)
Intermediate Care Unit		18 (12)
Other		11 (8)

* Multiple answers possible. Proportions can exceed 100%. SD = standard deviation; IQR = interquartile range.

**Table 2:** Type and timing of aneurysm treatment.

Aneurysm Treatment Characteristic	*n* Completed	Mean Estimate (SD), *n* (%)
**Proportion of aneurysms treated by endovascular coiling**	130	65% (26)
**Timing of aneurysm treatment**		
Proportion of aneurysm treatment <24 h from symptom onset †	145	65 (30)
Proportion of aneurysm treatment 24–48 h from symptom onset †	145	18 (16)
Proportion of aneurysm treatment >48 h from symptom onset †	145	8 (12)
Number of centers that treat the majority (>50%) of aneurysms in particular time window from symptom onset *	145	
Within 24 h from symptom onset		96 (66)
Within 24–48 h from symptom onset		7 (5)
Within 48–72 h from symptom onset		3 (2)

† Estimated mean percentages do not add up to 100%. Nine percent is unaccounted for. * Percentages do not add up to 100%. Not all centers treat a majority of aneurysms in a particular time window. SD = standard deviation.

**Table 3:** Type and timing of aneurysm treatment per geographical region.

Aneurysm treatment characteristic	Europe (EU) Mean % (SD)	United States (US) Mean % (SD)	Other * Mean % (SD)	*p*-Value
**Aneurysms treated endovascular**	70 (18)	72 (19)	51 (33)	EU vs. other *p* < 0.01; US vs. other *p* < 0.001
**Aneurysms treated <24 h ^†^**	67 (24)	77 (23)	46 (34)	EU vs. other *p* < 0.01; US vs. other *p* < 0.001
**Aneurysms treated ≥24 h ^†^**	30 (21)	22 (22)	30 (24)	0.109

* All non-US and non-EU centers are categorized as “other”. † Estimated mean percentages do not add up to 100%. *p*-values are calculated with ANOVA, and in case of a significant result they are calculated with a post-hoc multiple comparison Bonferroni test. Only significant *p*-values are reported. SD = standard deviation.

**Table 4:** Fluid management in aSAH.

Fluid Management Characteristic	*n* Completed	*n* (%)
**What is the goal of maintenance intravenous fluids in aneurysmal subarachnoid hemorrhage (aSAH) in your hospital?**	142	
Euvolemia		136 (96)
Hypervolemia		5 (3)
Other		1 (1)
**What goal of net fluid balance for aSAH patients do you use at your institution?**	124	
−500 mL/day		0
0 mL/day		66 (53)
+500 mL/day		51 (41)
+1 L/day		2 (2)
Other		5 (4)
**What goal of fluid intake for aSAH patients do you use at your institution?**	122	
1 L/day		11 (9)
2 L/day		66 (54)
3 L/day		0
>3.5 L/day		2 (2)
Other		43 (35)
**Does your center use clinical blood tests to guide fluid management of aSAH patients?**	141	
Yes		69 (49)
No		72 (51)
Which? *	69	
Troponin		30 (44)
B-type (or brain) natriuretic peptide		31 (45)
Neuron-specific enolase		3 (4)
Interleukin 6		2 (3)
Lactate		56 (81)
Others		9 (13)
**Which maintenance fluids do you use for aSAH patients in your hospital?** *	118	
0.9% saline		101 (86)
3% saline		1 (1)
5% human albumin		11 (9)
20% human albumin		3 (3)
25% human albumin		4 (3)
Synthetic colloid		1 (1)
Balanced solutions		59 (50)
Other		2 (2)
**Do you use advanced hemodynamic monitoring to guide fluid management at the intensive care unit?**	128	
Yes		108 (84)
No		20 (16)
** How often?**	105	
<10% of patients		18 (17)
10–25% of patients		40 (38)
>25% of patients		47 (45)
With what device? *	110	
Echocardiography/Inferior vena cava		85 (77)
Pulmonary artery catheter		11 (10)
Transpulmonary thermodilution		53 (48)
Other		29 (26)

* Multiple answers possible. The sample total can exceed *n* = 145.

**Table 5:** Regional differences in vasospasm and DCI management.

DCI Management Characteristic	*n* Completed	Europe (EU) *n*, (%)	United States (US) *n*, (%)	Other * n, (%)	*p*-Value
**Do you routinely administer nimodipine to patients with aneurysmal subarachnoid hemorrhage (aSAH)?**	139	35 (97)	62 (100)	39 (95)	0.238
**Do you use induced hypertension when aSAH patients develop delayed cerebral ischemia (DCI)?**	141	32 (87)	62 (100)	34 (81)	US vs. other *p* < 0.05; US vs. EU *p* < 0.05
**Do you induce hypervolemia when aSAH patients develop DCI?**	142	6 (16)	19 (30)	12 (29)	0.280
**Do you induce hemodilution when aSAH patients develop DCI?**	123	2 (6)	6 (12)	6 (16)	0.391
**Does your center use biomarkers or laboratory tests to guide DCI management?**	124	9 (26)	14 (26)	5 (14)	0.289
**Do you perform endovascular therapies when vasospasm is shown on cerebral angiography?**	124	25 (74)	48 (91)	22 (60)	US vs. other *p* < 0.05

* All non-US and non-EU centers are categorized as “other”. *p*-values are calculated with chi-squared test, and in case of a significant result, a post-hoc multiple comparison Bonferroni test. Only significant *p*-values are reported.

## Data Availability

The data presented in this study are available on request from the corresponding author. The data elements collected are presented in the “Provider Profiling Questionnaire (See: Electronic Supplementary File S1).

## Supplementary Materials

The Provider Profiling Questionnaire is available online at https://www.mdpi.com/2077-0383/10/4/762/s1. File S1: Insider study.
