# Supplementary material for: International Practice Variability in Treatment of Aneurysmal Subarachnoid Hemorrhage
Source: J Clin Med. 2021 Feb 14;10(4):762. doi: 10.3390/jcm10040762 (PMC7917699; doi:10.3390/jcm10040762)
Supplement: Supplementary file 1 [file jcm-10-00762-s001.pdf]

INSIDER STUDY

PIs: Eusebia Calvillo (The Johns Hopkins Univeristy), Sherry H-Y Chou (University of Pittsburgh), Nima Etmiman (Medical Faculty Mannheim, University of Heidelberg, Germany), Nerissa Ko (UCSF), Judy Huang (The Johns Hopkins University), Hester F Lingsma (Erasmus MC University Medical Center Rotterdam, The Netherlands), R Loch Macdonald (University of Toronto), Renee H Martin (Medical University of South Carolina), Jose I Suarez (The Johns Hopkins University), Mathieu van der Jagt (Erasmus MC University Medical Center Rotterdam, The Netherlands), Chethan P Venkatasubba Rao (Baylor College of Medicine), Mervyn D I Vergouwen (University Medical Center Utrecht, The Netherlands).

We are approaching you about participating in the INSIDER (***IN***ternational ***S***ubarachno***ID*** Hemorrhage Comparative ***E***ffectiveness ***R***esearch Alliance) study. This is a seven-year prospective, observational, international, and multicenter study to determine the effect of practice variability on outcome after aneurysmal subarachnoid hemorrhage (aSAH), answer clinically relevant research questions, develop an outcome prediction model, and build a knowledge treasury to support future SAH research. We are seeking funding from NINDS/NIH to support the INSIDER study and are planning on including in the grant budget monetary compensation to cover the expenses of participating sites for data collection for this observational study.

Please complete the questionnaire to indicate your interest in participating. Your response will help us understand the infrastructure available in your institution to support the study, and your management practice of aSAH patients. We need your input to generate important preliminary data for the NINDS grant application. Thank you for taking the time to complete the survey. We anticipate that it will take you about 25-30 minutes to complete this survey. If you decide to participate please do so by Friday April 26, 2019.

General Characteristics of your Institution

1. Name of institution:____
2. Contact person:____
3. Contact e-mail address:____
4. Would you be interested in participating in the INSIDER Study? Yes/No
5. if no stop
6. Type of institution
    a. Academic Center
    b. Private Non-Academic
    c. Public Non-Academic
    d. Other____
7. Number of beds in your institution:
    a. <250
    b. 250 – 500
    c. 500 – 750
    d. 750 – 1000
    e. > 1000
8. Population of the city where your hospital is located:
    a. < 100,000
    b. 100,000 – 250,000
    c. 250,000 – 500,000
    d. 500,000 – 750,000
    e. 750,000 – 1,000,000
    f. > 1,000,000
9. How many patients per year do you see with aSAH at your center?
    a. 0-40

b. 40-60
c. 60-100
d. 100-150
e. 150-200
f. >200

10. What percentage of patients with aSAH present directly to your hospital (as opposed to coming in as transfer from another medical facility)?
11. Where are aSAH patients admitted? (check all that apply)
    a. Dedicated Neuro ICU
    b. Surgical ICU
    c. Medical ICU
    d. Medical-Surgical ICU
    e. Intermediate Care Unit
    f. Other:____
12. Number of ICU beds in your hospital: ___________________________________
13. Number of neuroICU beds in your hospital: ______________________________
14. Do you have clinical research coordinators (CRC) / research nurses at your institution? Yes/No
15. How many full-time CRCs/nurses are at your program?
16. Does your IRB have provisions to use a central IRB? Yes/No
17. What percentage of aSAH patients have their aneurysm repaired by endovascular coiling?
18. What proportion of aSAH patients have their aneurysm repaired within 24 h from symptom onset?
19. What proportion of aSAH patients have their aneurysm repaired within 24-48 h from symptom onset?
20. What proportion of aSAH patients have their aneurysm repaired within 48-72 h from symptom onset?
21. Do you regularly perform MRI on aSAH patients? Yes/No
22. If yes to question 25, specify when:
    a. during hospitalization
    b. within 3 months after discharge
    c. 3-6 months after discharge
    d. other
23. What type of MRI is available at your center? (check all that apply)
    a. 1.5T MRI
    b. 3T MRI
    c. fMRI
    d. Other
24. What is the goal of maintenance intravenous fluids in aSAH in your hospital?
    a. Euvolemia
    b. hypervolemia
    c. other
25. Does your center use clinical blood tests to guide fluid management of aSAH patients (please check all that apply)?
    a. Troponin
    b. B-type (or brain) natriuretic peptide (BNP)
    c. Neuron specific enolase (NSE)
    d. Interleukin 6 (IL-6)
    e. lactate
    f. Others (please specify)
26. If Yes to question 25, on what post-bleed days do you check these labs? (day of SAH onset = post bleed day 1)

27. Which maintenance fluids do you use for aSAH patients in your hospital? (please check all that apply)
    a. 0.9% saline
    b. 5% human albumin
    c. 25% human albumin
    d. 20% human albumin
    e. synthetic colloid
    f. balanced solutions (e.g. Plasmalyte, Lactated Ringers)
    g. other
28. What goal of fluid intake for aSAH patients do you use at your institution?
    a. 1 L/day
    b. 2 Lts/day
    c. 3 Lts/day
    d. >3.5 L/day
    e. Other
29. What goal of net fluid balance for aSAH patients do you use at your institution?
    a. -500 ml/day
    b. 0 ml/day
    c. +500 ml/day
    d. +1L/day
    e. Other
30. Do you routinely administer nimodipine to patients with aSAH? Yes/No
31. Do you use induced hypertension when aSAH patients develop delayed cerebral ischemia (DCI)? Yes/No
32. Do you induce hypervolemia when aSAH patients develop DCI? Yes/No
33. Do you induce hemodilution when aSAH patients develop DCI? Yes/No
34. Does your center use any biomarkers/lab tests to guide DCI management such as initiation or duration of therapies such as induced hypertension, HHH, and endovascular therapies in SAH? Yes/No
35. If Yes to question 34, please specify what lab tests you use?

36. Do you administer endovascular therapies (angioplasty, intra-arterial infusions of vasodilators) when vasospasm is shown on cerebral angiography? Yes/No
37. Do you collect and/or store any biospecimens (blood, CSF, genomic) at your institution? Yes/No
38. If yes to question 37: What type of samples do you collect? (please check all that apply)
    a. Blood
    b. CSF
    c. DNA
    d. Other
39. Do you have access to a centrifuge and laboratory space to perform basic biospecimen processing (centrifugation, pipetting/aliquoting): Yes/No
40. Do you have access to a -80C deep freezer for short term (up to 3 to 6 months) or long term biospecimen storage? Yes/No,
41. If Yes to question 40, please indicate:
    a. Short term
    b. Long term.
42. If yes to question 40, are you interested in contributing samples or data to the biomarker sub-study of INSIDER? Yes/No
